# Supplementary material for: Mental health is positively associated with biodiversity in Canadian cities
Source: Commun Earth Environ. 2024 Jun 11;5(1):310. doi: 10.1038/s43247-024-01482-9 (PMC11166573; doi:10.1038/s43247-024-01482-9)
Supplement: Supplementary file 2 — Supplementary Information [file 43247_2024_1482_MOESM2_ESM.pdf]

Supplementary Materials for

Mental health is positively associated with biodiversity in Canadian cities

Supplementary Methods

Standardized estimates of bird species richness and diversity

Given eBird surveys occur at any time of year and checklists can vary in length, we created a generalized linear mixed model (GLMM) to standardize species diversity estimates. We created two models with species richness (with a Poisson error structure) and Shannon diversity (with a Gaussian error structure) as response variables. We included Julian date and checklist duration as fixed effects and year and locality as random effects. Models were run for each Canadian Census Metropolitan Areas (CMA) separately (models would not converge with all data included). We then used these models to predict species richness and diversity for a 120 min checklist on June 5 (including breeding season but excluding spring migrants).

Biases in eBird checklists

A previous study in Boston and Phoenix, U.S. found that more checklists were submitted in areas with higher income and higher proportion of white residents (1). Thus, we explored potential SES biases in eBird sampling effort in Canadian CMA postal codes in our dataset. We quantified the relationship between the number of checklists collected in a postal code and a neighborhood-level index of marginalization. The Canadian Marginalization Index from the Canadian Urban Environmental Health Research Consortium (CANUE) was available at the postal code level, calculated for 2016, and describes material deprivation, residential instability, dependency, and ethnic concentration quantified in continuous values and categorized in quintiles (2). Using a generalized linear model with a Poisson error structure we found little evidence of a relationship between metrics of marginalization and number of checklists ($R^2 < 0.02$).

People often travel from where they live to carry out eBird checklists. As such, we did not expect there to be a relationship between population density and the number of checklists. For example, in Ottawa, localities with the greatest number of checklists include Richmond Sewage Lagoons and Rockcliffe Airport Woods, both areas with low population density. We expect that rarefaction would control for any effect of uneven sampling at a large scale.

Effect modifier

To account for evidence suggesting that metrics of SES can affect both biodiversity and mental health (3, 4), we stratified our analysis by neighbourhood marginalization. We explored the relationship between metrics of biodiversity and indicators of mental health in neighborhoods with low and high values of Canadian Marginalization Index from CANUE. Because there are four dimensions of marginalization (instability, deprivation, dependency, and ethnic concentration), to select the dimension with the highest likelihood of modifying the relationship between mental health and biodiversity, we explored the relationship between species diversity of birds and trees and each marginalization dimension. We fitted 16 GLMs, 8 with a Poisson error structure with species richness of birds or trees as the response and 8 with a Gaussian error structure with Shannon diversity of birds or trees as the response. We selected the "instability" marginalization dimension, as these models had the best fit with both tree and bird species diversity (tree species richness $R^2 = 0.4$). We then considered high marginalization as values in the top two quintiles of instability among Canadian postal codes (values of 4 or 5).

Analysis

To explore the effect of environmental variables on mental health metrics we used generalized additive models (GAMs) for very large datasets in the *bam* package (5). The GAM

approach is flexible, including each covariate as a smooth function that allows for non-linear relationships, while avoiding overfitting (6). Because model selection likely would not remove entire smooth terms from a model, analogous to GAM, *bam* and allows for the specification of a "select" option that assesses the optimal number of knots in each smoother term. We fit models with the bam default method fREML (fast REML), and discretized covariates for efficiency (7). To further avoid fitting overly complex smoother terms, we capped the maximum knots fit by any model at five. Because our goal was to understand relationships between biodiversity and mental health, rather than to understand average Canadian wellbeing, we use unweighted CCHS data throughout our analyses (8).

We fitted two sets of logistic GAMs (binomial models with a logit link) using the following response variables: 1) high/low self-rated stress and 2) high/low self-rated mental health. To control for unexplained spatial variability and nonlinear temporal variability in mental health, each model had a basic structure containing CMA as a random effect, as well as a continuous smoother fit to CCHS survey year and postal code area. For each response variable we fit three models: 1) only biodiversity and bluespace/greenspace variables; 2) adding in socio-demographic characteristics (age, sex, marital status, income, education, ethnicity, and immigration status); and 3) adding in health behaviours. All continuous variables were centred and scaled by subtracting the mean and dividing by the standard deviation. All variables apart from survey year and CMA were included as parametric terms (i.e., were included in the model without a smoother) to allow comparison of the relative influence of categorical and continuous variables and penalizing models against overfitting complex smoothers. Different amounts of missing data meant that each of these models contained a different sample size of complete cases.

For the first model, we chose the most parsimonious combination of NDVI, tree and bird diversity metrics, and distance to and density of bluespace/greenspace by fitting models with different combinations and selecting the model with the lowest AIC for each response variable (24 possible models). To assess collinearity among variables, we computed a Spearman's correlation matrix. For variables with a correlation coefficient (R) greater than 0.7 (9) we included one of each covariate in a separate model (where it was the only term) and chose the covariate with the model that resulted in the lowest Akaike's Information Criterion (AIC). Each *BAM* fit was further assessed for concurvity (the GAM equivalent of multicollinearity; 10), which can persist among smoothed terms even in the absence of strongly collinear variables. When high concurvity was detected for survey year (worst case concurvity of >0.8 in the *concurvity* function), the number of knots associated with the smoother term for survey year was iteratively reduced until concurvity decreased below 0.8. If concurvity persisted, survey year was converted to a parametric term.

In the third model set (with sociodemographic characteristics and health behaviors) we found evidence of complete separation (response variable separates the categorical predictor variables perfectly) due to large amounts of missing CCHS data, leading to large parameter and confidence interval estimates. To ensure robustness in our parameter estimates, we compared the results from models where missing values were imputed to the median and missing values were imputed with multiple imputation by chained equations via the *mice* R package following the approach outlined in (11), where we performed 50 imputations and used our full model equation as our imputation structure. We found little difference in our results using these methods, thus use the imputation method in all further analysis.

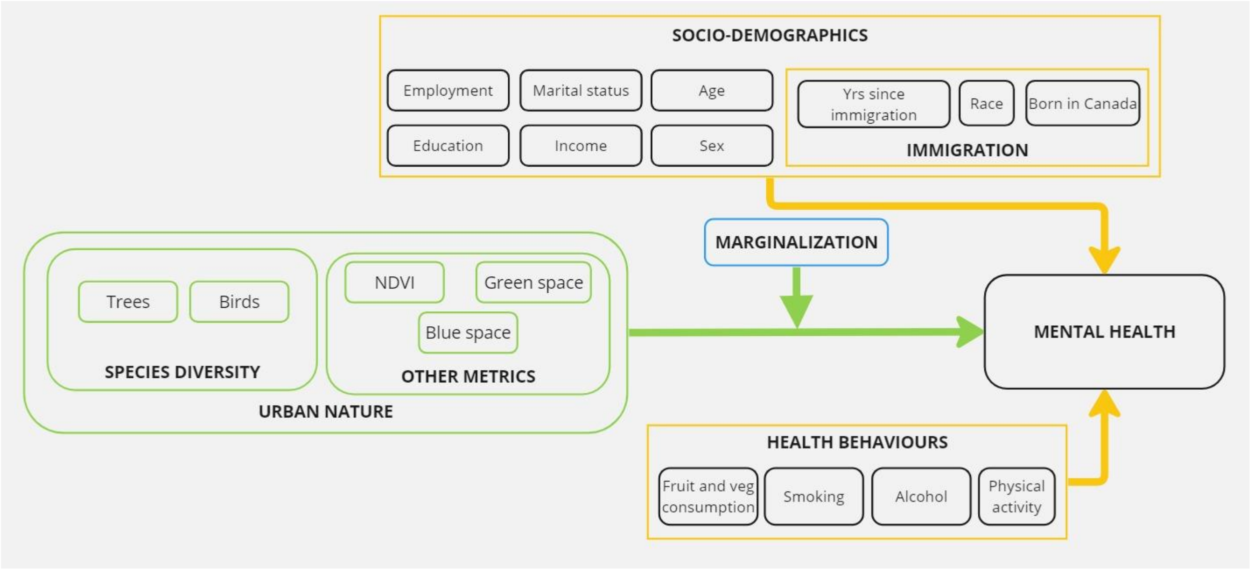

Figure S1.

Conceptual model showing the potential relationship between mental health (response variable), nature-related variables (dependent variables, green including primary exposure 'species diversity'), health behaviors and socio-demographics (covariates, yellow), and socio-economic marginalization (moderator, blue) used to guide the analysis.

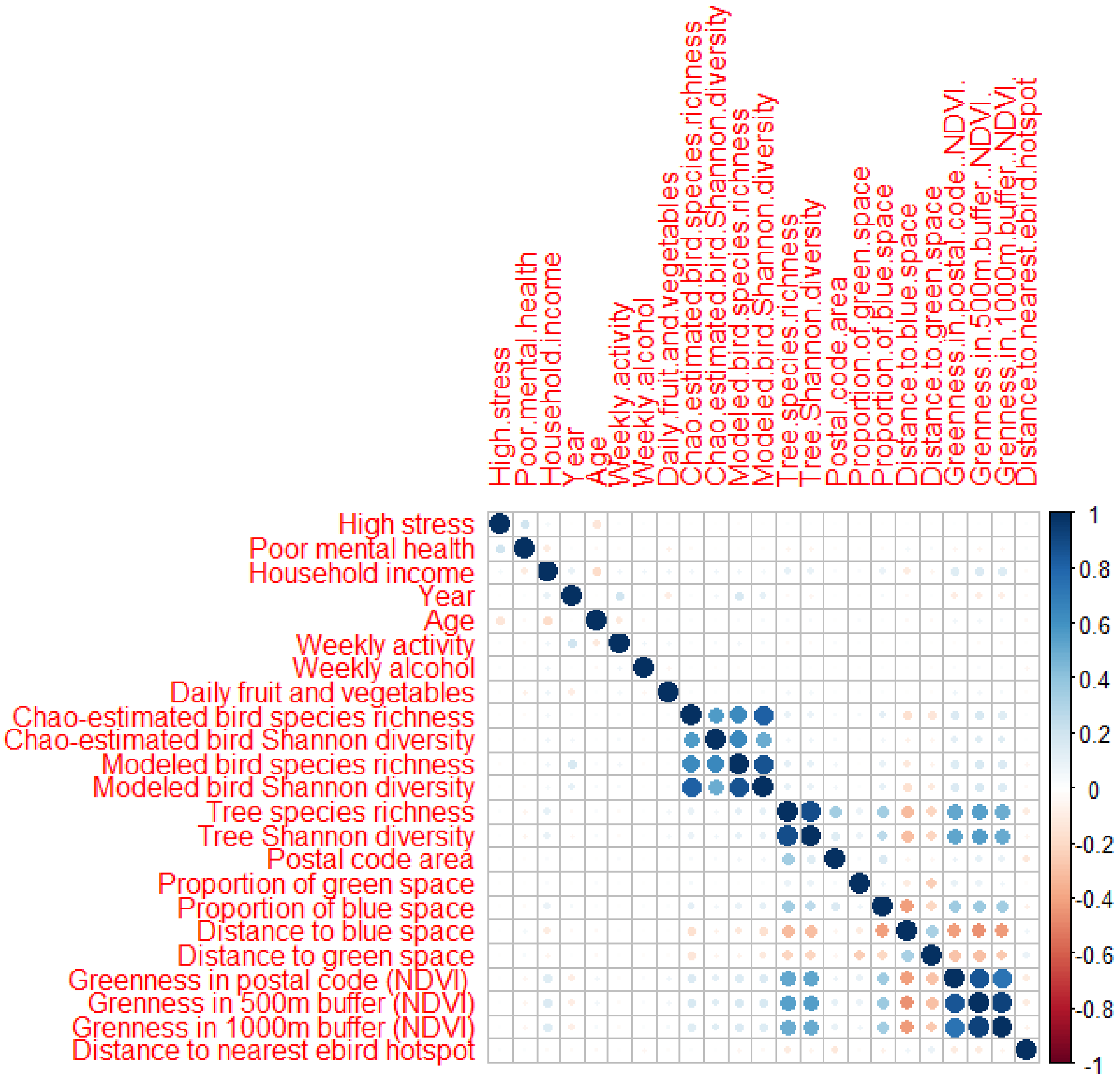

Figure S2.

Pairwise Spearman's correlations between among all continuous variables (dependent and independent variables and potential confounders). Any variables with an *R*>= 0.5 were not included in the same model. The results presented were generated using the full data set – the same pairs of variables had *R*>= 0.7 when data were stratified by high and low marginalization.

Table S1.

Differences in mean and standard deviation (SD) of all variables in the entire set of CCHS respondents >18 years of age within Canadian Census Metropolitan Areas (overall) compared to those living in postal codes with sufficient eBird data to be included in our analysis (within sample). Data were stratified by high marginalization (top two quintiles of instability of Canadian Urban Environmental Health Research Consortium's Canadian Marginalization Index) and low marginalization (bottom 3 quintiles of instability).

	**All data**		**High marginalization**		**Low marginalization**	
**Variable**	**Overall mean ± SD**	**Within sample mean ± SD**	**Overall mean ± SD**	**Within sample mean ± SD**	**Overall mean ± SD**	**Within sample mean ± SD**
Household income (1-15)	10.1 ± 3.5	10.58 ± 3.59*	10.16 ± 3.54	10.54 ± 3.64*	10.04 ± 3.45	10.61 ± 3.52*
Age (years)	51.86 ± 19.01	53.8 ± 19.01*	49.38 ± 18.66	51.26 ± 18.8*	54.58 ± 19.02	56.95 ± 18.79*
Daily Fruit and Vegetable Consumption	4.63 ± 2.58	4.38 ± 2.5†	4.58 ± 2.57	4.33 ± 2.48†	4.69 ± 2.59	4.43 ± 2.5†
Weekly Alcohol Consumption	4.31 ± 7.18	4.11 ± 6.79†	3.87 ± 6.73	3.73 ± 6.39†	4.76 ± 7.59	4.54 ± 7.19†
Year	2013.87 ± 4.38	2017.4 ± 3.14*	2013.87 ± 4.37	2017.33 ± 3.18*	2013.84 ± 4.38	2017.48 ± 3.08*
Weekly Active Time (hours)	6.81 ± 19.16	9.05 ± 21.68*	6.82 ± 19.21	9 ± 22.03*	6.79 ± 19.11	9.11 ± 21.21*
Chao-Estimated Bird Species Richness	30.32 ± 16.48	30.32 ± 16.48	30.35 ± 16.7	30.35 ± 16.7	30.29 ± 16.22	30.29 ± 16.22
Self-rated High Life Stress (0/1)	0.21 ± 0.41	0.2 ± 0.4†	0.22 ± 0.42	0.21 ± 0.41†	0.2 ± 0.4	0.19 ± 0.4†
Self-rated Poor Mental Health (0/1)	0.07 ± 0.26	0.08 ± 0.27*	0.07 ± 0.26	0.08 ± 0.28*	0.07 ± 0.25	0.07 ± 0.26
Chao-Estimated Bird Shannon Diversity	2.39 ± 0.58	2.39 ± 0.58	2.41 ± 0.58	2.41 ± 0.58	2.36 ± 0.57	2.36 ± 0.57
Modeled Bird Species Richness	1.51 ± 0.36	1.51 ± 0.36	1.51 ± 0.36	1.51 ± 0.36	1.5 ± 0.36	1.5 ± 0.36

Modeled Bird Shannon Diversity	8.29 ± 3.62	8.29 ± 3.62	8.26 ± 3.68	8.26 ± 3.68	8.32 ± 3.53	8.32 ± 3.53
Tree Species Richness	7.12 ± 7.49	6.83 ± 7.41†	5.72 ± 6.54	5.84 ± 6.88*	8.66 ± 8.14	8.05 ± 7.84†
Tree Shannon Diversity	1.08 ± 0.92	1.04 ± 0.9†	0.93 ± 0.88	0.94 ± 0.88*	1.25 ± 0.92	1.17 ± 0.91†
Postal Code Area (km^2)	0.13 ± 0.91	0.13 ± 1.01	0.07 ± 0.64	0.1 ± 0.93*	0.21 ± 1.13	0.17 ± 1.11†
Proportion of blue space	0.01 ± 0.06	0.01 ± 0.06	0.01 ± 0.05	0.01 ± 0.05	0.02 ± 0.07	0.02 ± 0.07
Proportion of green space	0.07 ± 0.19	0.06 ± 0.17†	0.04 ± 0.14	0.04 ± 0.14	0.1 ± 0.22	0.08 ± 0.2†
Distance to blue space (km)	0.28 ± 0.22	0.28 ± 0.21	0.3 ± 0.22	0.29 ± 0.21†	0.25 ± 0.21	0.26 ± 0.22*
Distance to green space (km)	0.51 ± 0.38	0.51 ± 0.36	0.57 ± 0.39	0.56 ± 0.37†	0.45 ± 0.36	0.45 ± 0.34
Greenness in postal code (NDVI)	0.43 ± 0.23	0.29 ± 0.27†	0.41 ± 0.22	0.28 ± 0.26†	0.46 ± 0.24	0.3 ± 0.28
Greenness at 500m buffer (NDVI)	0.44 ± 0.23	0.29 ± 0.27†	0.42 ± 0.22	0.29 ± 0.26†	0.46 ± 0.24	0.3 ± 0.28
Greenness at 1000m buffer (NDVI)	0.44 ± 0.23	0.29 ± 0.27†	0.43 ± 0.22	0.29 ± 0.27†	0.46 ± 0.24	0.3 ± 0.28
Distance to nearest eBird hotspot	425.85 ± 278.55	425.85 ± 278.55	0.45 ± 0.28	0.45 ± 0.28	0.39 ± 0.27	0.39 ± 0.27

* Within sample means are greater than overall means
† Within sample means are less than overall means

Table S2.

Model selection tables for biodiversity and greenness covariates for self-rated mental health and self-rated stress. Greenness variables include normalized difference vegetation index (NDVI) within a postal code (ndvi), NDVI within 500 meters from a postal code edge (ndvi500), and NDVI within 1000 metres from a postal code edge (ndvi1000). Tree diversity variables include tree species richness and tree Shannon diversity. All models included a smoother for the year term (k=5 with smoother selection by REML) and a random effect of CMA. Edf = empirical degrees of freedom from a generalized additive model used to calculate Akaike Information Criterion (AIC).

Mental health

NDVI varriable	Tree diversity variable	Bird diversity variable	edf	AIC	ΔAIC	Akaike weight
**ndvi500**	**Tree sp richness**	**Modelled Shannon diversity**	**37.49**	**26185.90**	**0.00**	**0.354**
ndvi1000	Tree sp richness	Modelled Shannon diversity	37.38	26186.91	1.00	0.214
ndvi500	Tree Shannon diversity	Modelled Shannon diversity	37.63	26187.47	1.57	0.162
ndvi1000	Tree Shannon diversity	Modelled Shannon diversity	37.53	26188.49	2.58	0.097
ndvi	Tree sp richness	Modelled Shannon diversity	36.98	26188.59	2.68	0.093
ndvi	Tree Shannon diversity	Modelled Shannon diversity	37.13	26190.21	4.31	0.041
ndvi500	Tree sp richness	Modelled sp richness	37.25	26193.29	7.38	0.009
ndvi1000	Tree sp richness	Modelled sp richness	37.08	26194.10	8.20	0.006
ndvi500	Tree sp richness	Chao estimated sp richess	36.54	26194.82	8.92	0.004
ndvi500	Tree Shannon diversity	Modelled sp richness	37.39	26194.96	9.05	0.004
ndvi	Tree sp richness	Modelled sp richness	36.65	26195.17	9.26	0.003
ndvi1000	Tree sp richness	Chao estimated sp richess	36.37	26195.58	9.68	0.003
ndvi1000	Tree sp richness	Modelled sp richness	37.22	26195.78	9.87	0.003
ndvi500	Tree Shannon diversity	Chao estimated sp richess	36.69	26196.44	10.54	0.002
ndvi	Tree sp richness	Chao estimated sp richess	36.01	26196.54	10.64	0.002
ndvi	Tree Shannon diversity	Modelled sp richness	36.78	26196.89	10.98	0.001
ndvi1000	Tree Shannon diversity	Chao estimated sp richess	36.52	26197.20	11.30	0.001
ndvi	Tree Shannon diversity	Chao estimated sp richess	36.15	26198.20	12.30	0.001

ndvi500	Tree sp richness	Chao estimated Shannon diversity	36.70	26200.55	14.64	0.000
ndvi1000	Tree sp richness	Chao estimated Shannon diversity	36.52	26201.42	15.52	0.000
ndvi500	Tree Shannon diversity	Chao estimated Shannon diversity	36.85	26202.27	16.36	0.000
ndvi	Tree sp richness	Chao estimated Shannon diversity	36.12	26202.47	16.57	0.000
ndvi1000	Tree Shannon diversity	Chao estimated Shannon diversity	36.67	26203.15	17.25	0.000
ndvi	Tree Shannon diversity	Chao estimated Shannon diversity	36.26	26204.24	18.34	0.000

Life Stress

**NDVI varriable**	**Tree diversity variable**	**Bird diversity variable**	**edf**	**AIC**	**ΔAIC**	**Akaike weight**
**ndvi**	**Tree Shannon diversity**	**Modelled sp richness**	**32.563**	**49023.8**	**0**	**0.12943**
ndvi	Tree sp richness	Modelled sp richness	32.5305	49023.8	0.04288	0.126684
ndvi	Tree Shannon diversity	Modelled Shannon diversity	32.5845	49024	0.21703	0.11612
ndvi	Tree sp richness	Modelled Shannon diversity	32.5526	49024.1	0.25121	0.114153
ndvi	Tree Shannon diversity	Chao estimated sp richess	32.5239	49025.6	1.83432	0.051727
ndvi	Tree sp richness	Chao estimated sp richess	32.488	49025.7	1.88281	0.050488
ndvi500	Tree Shannon diversity	Modelled sp richness	32.5717	49025.9	2.05344	0.046359
ndvi500	Tree sp richness	Modelled sp richness	32.541	49025.9	2.09014	0.045516
ndvi500	Tree Shannon diversity	Modelled Shannon diversity	32.5914	49026.1	2.26473	0.041711
ndvi500	Tree sp richness	Modelled Shannon diversity	32.5614	49026.1	2.29289	0.041128
ndvi	Tree Shannon diversity	Chao estimated Shannon diversity	32.4449	49027	3.20457	0.026072
ndvi1000	Tree sp richness	Modelled sp richness	32.5348	49027	3.22762	0.025773
ndvi1000	Tree sp richness	Modelled sp richness	32.5057	49027.1	3.25721	0.025395
ndvi1000	Tree Shannon diversity	Modelled Shannon diversity	32.5546	49027.2	3.41171	0.023507
ndvi1000	Tree sp richness	Modelled Shannon diversity	32.5264	49027.2	3.43314	0.023256
ndvi500	Tree Shannon diversity	Chao estimated sp richess	32.5339	49027.7	3.88142	0.018586
ndvi500	Tree sp richness	Chao estimated sp richess	32.4998	49027.7	3.92307	0.018203
ndvi1000	Tree Shannon diversity	Chao estimated sp richess	32.4945	49028.9	5.0511	0.010356
ndvi1000	Tree sp richness	Chao estimated sp richess	32.462	49028.9	5.08484	0.010183
ndvi500	Tree Shannon diversity	Chao estimated Shannon diversity	32.4572	49029	5.21647	0.009534
ndvi500	Tree sp richness	Chao estimated Shannon diversity	32.4244	49029.1	5.25222	0.009365

ndvi1000	Tree Shannon diversity	Chao estimated Shannon diversity	32.4191	49030.1	6.31598	0.005502
ndvi1000	Tree sp richness	Chao estimated Shannon diversity	32.388	49030.1	6.34495	0.005423

Table S3.

Details of generalized additive models exploring the relationship between self-rated mental health and stress including only biodiversity and green and blue space variables (biodiversity), adding in socio-demographic variables (+ sociodem), and adding socio-demographic and health behaviours (+ health behaviour). This includes the degrees of freedom (df), X^2, and P of the random effect for Canadian Metropolitan Area (CMA), Akaike Information Criterion (AIC), adjusted R^2, deviance explained and n for each model. In the model including biodiversity, socio-demographic, and health behaviour variables missing data were interpolated using a multivariate imputation by chained equations algorithm. We used all data, only postal codes with the top two quintiles of the Canadian Urban Environmental Health Research Consortium's Canadian marginalization index 'instability' variable (high marginalization), and only postal codes with the bottom three quintiles of instability (low marginalization).

			Random effect for CMA						
	Model	Data	df	X^2	P	AIC	R^2_{adj}	Deviance explained	N
Mental health	Biodiversity	All	25.56	143.8	<0.001	26188	0.0078	1.52%	47623
	+ sociodem	All	26.87	188.64	<0.001	24365.3	0.042	6.55%	46522
	+ health behaviour	All	26.77	188.02	<0.001	24156.2	0.049	7.43%	46522
	Biodiversity	Low marginalization	25.46	136.38	<0.001	11134.8	0.012	2.38%	21278
	+ sociodem	Low marginalization	25.81	146.6	<0.001	10400.1	0.047	7.53%	20811
	+ health behaviour	Low marginalization	25.38	132.24	<0.001	10346.7	0.051	8.17%	20811
	Biodiversity	High marginalization	8.64	18.18	0.005	149923	0.0005	1.00%	26219
	+ sociodem	High marginalization	12.68	48.93	<0.001	13911	0.041	6.22%	25586
	+ health behaviour	High marginalization	13.02	53.47	<0.001	13769.4	0.05	7.38%	25586
Stress	Biodiversity	All	22.56	102.64	<0.001	49025.9	0.0036	0.44%	48693
	+ sociodem	All	214.49	39.24	<0.001	46355.2	0.034	3.72%	47543
	+ health behaviour	All	15.38	42.03	<0.001	46207.2	0.038	4.07%	47543
	Biodiversity	Low marginalization	19.54	61.44	<0.001	21361.7	0.005	0.69%	21771
	+ sociodem	Low marginalization	19.54	61.44	0.0011	20016.5	0.045	4.95%	21277
	+ health behaviour	Low marginalization	13.07	27.64	0.00058	19965.5	0.048	5.30%	21277
	Biodiversity	High marginalization	9.98	26.73	0.00018	27542.6	0.0016	0.26%	26793

	+ sociodem	High marginalization	4.34	7.47	0.05	27125.6	0.026		2.85%	26138
	+ health behaviour	High marginalization	3.68	5.75	0.089	26154.8	0.03		3.19%	26138

Table S4.

Comparison of parameter estimates (PE) +/- standard error (SE) and p-values (P) from generalized additive models exploring the relationship between self-rated mental health and variables relating to biodiversity, socio-demographics, and health behaviours. Because of high numbers of missing values, we compared two methods of dealing with missing variables: imputed missing values using multiple imputation by chained equations (MICE) and replacing missing data with the median (Median).

Variable	MICE		Median	
	PE ± SE	P	PE ± SE	P
Intercept	-1.83 ± 0.15	0.00	-1.78 ± 0.15	0.00
Tree sp richness	-0.06 ± 0.03	0.04	-0.06 ± 0.03	0.04
Distance to eBird locality	0.00 ± 0.02	0.91	0.00 ± 0.02	0.88
Bird Shannon diversity	-0.07 ± 0.02	0.00	-0.05 ± 0.02	0.02
NDVI (500m buffer)	-0.03 ± 0.04	0.57	-0.06 ± 0.05	0.17
Year	0.26 ± 0.06	0.00	0.32 ± 0.06	0.00
Distance to blue space	0.04 ± 0.02	0.08	0.04 ± 0.02	0.07
Distance to green space	-0.01 ± 0.02	0.66	-0.01 ± 0.02	0.68
Prop of blue space	-0.01 ± 0.02	0.52	-0.01 ± 0.02	0.59
Prop of green space	-0.03 ± 0.03	0.16	-0.03 ± 0.03	0.18
Postal code area	0.00 ± 0.02	0.90	0.00 ± 0.02	0.88
Common-law	0.20 ± 0.07	0.01	0.20 ± 0.07	0.01
Never married	0.11 ± 0.08	0.15	0.11 ± 0.08	0.14
Separated	0.64 ± 0.09	0.00	0.65 ± 0.09	0.00
Divorced	0.48 ± 0.06	0.00	0.49 ± 0.06	0.00
Widowed	0.47 ± 0.05	0.00	0.48 ± 0.05	0.00
Unk marital status	0.86 ± 0.32	0.01	0.87 ± 0.32	0.01
Employed	-0.47 ± 0.05	0.00	-0.46 ± 0.05	0.00
Unk employment status	-0.25 ± 0.07	0.00	-0.25 ± 0.07	0.00
White	0.21 ± 0.10	0.03	0.22 ± 0.10	0.03
Unk ethnicity	0.26 ± 0.10	0.01	0.25 ± 0.11	0.02
Non-immigrant, non-White	0.42 ± 0.34	0.22	0.42 ± 0.34	0.22
Immigrant, White, <10 yrs	-6.15 ± 50.77	0.90	-6.21 ± 50.74	0.90
Unk immigration status	-0.05 ± 0.09	0.55	-0.05 ± 0.09	0.54
Household income	-0.24 ± 0.02	0.00	-0.26 ± 0.02	0.00
High school education	-0.37 ± 0.06	0.00	-0.37 ± 0.06	0.00
Post-secondary education	-0.41 ± 0.06	0.00	-0.42 ± 0.06	0.00
Unk education status	-0.41 ± 0.09	0.00	-0.44 ± 0.09	0.00

Female	0.12 ± 0.04	0.00	0.11 ± 0.04	0.00
Age	-0.36 ± 0.03	0.00	-0.37 ± 0.03	0.00
Weekly activity	-0.03 ± 0.02	0.07	-0.04 ± 0.02	0.05
Has not quit smoking	0.43 ± 0.15	0.01	0.43 ± 0.15	0.00
Unk smoking quit status	0.07 ± 0.38	0.86	0.11 ± 0.38	0.77
Never smoked	-0.24 ± 0.05	0.00	-0.24 ± 0.05	0.00
Occasional smoker	-0.31 ± 0.09	0.00	-0.31 ± 0.09	0.00
Non-smoker	-0.66 ± 0.05	0.00	-0.67 ± 0.05	0.00
Unk smoking frequency	-0.59 ± 0.60	0.33	-0.59 ± 0.60	0.33
Weekly alcohol	0.00 ± 0.02	0.80	0.02 ± 0.02	0.26
Weekly fruit and veg	-0.13 ± 0.02	0.00	-0.05 ± 0.01	0.00

Table S5.

Comparison of parameter estimates (PE) +/- standard error (SE) and p-values (P) from generalized additive models exploring the relationship between self-rated stress and variables relating to biodiversity, socio-demographics, and health behaviours. Because of high numbers of missing values, we compared two methods of dealing with missing variables: imputed missing values using multiple imputation by chained equations (MICE) and replacing missing data with the median (Median).

	MICE		Median	
Variable				
	PE ± SE	P	PE ± SE	P
Intercept	-1.70 ± 0.09	0.00	-1.70 ± 0.10	0.00
Tree sp richness	0.01 ± 0.01	0.62	0.01 ± 0.01	0.73
Distance to eBird locality	0.01 ± 0.01	0.59	0.01 ± 0.01	0.57
Bird Shannon diversity	-0.01 ± 0.01	0.39	-0.01 ± 0.01	0.27
NDVI (500m buffer)	0.08 ± 0.03	0.00	0.09 ± 0.03	0.00
Year	0.00 ± 0.04	0.96	-0.01 ± 0.04	0.72
Distance to blue space	0.01 ± 0.01	0.33	0.01 ± 0.01	0.34
Distance to green space	-0.01 ± 0.01	0.40	-0.01 ± 0.01	0.42
Prop of blue space	-0.02 ± 0.01	0.23	-0.02 ± 0.01	0.23
Prop of green space	-0.01 ± 0.01	0.50	-0.01 ± 0.01	0.49
Postal code area	-0.01 ± 0.01	0.56	-0.01 ± 0.01	0.56
Common-law	0.13 ± 0.04	0.00	0.13 ± 0.04	0.00
Never married	-0.15 ± 0.06	0.01	-0.15 ± 0.06	0.01
Separated	0.33 ± 0.06	0.00	0.33 ± 0.06	0.00
Divorced	0.19 ± 0.04	0.00	0.20 ± 0.04	0.00
Widowed	-0.03 ± 0.03	0.42	-0.02 ± 0.03	0.46
Unk marital status	0.46 ± 0.26	0.07	0.48 ± 0.26	0.06
Employed	0.54 ± 0.03	0.00	0.54 ± 0.03	0.00
Unk employment status	-0.02 ± 0.05	0.65	-0.03 ± 0.05	0.55
White	0.24 ± 0.06	0.00	0.24 ± 0.06	0.00
Unk ethnicity	0.32 ± 0.06	0.00	0.32 ± 0.07	0.00
Non-immigrant, non-White	0.15 ± 0.24	0.53	0.15 ± 0.24	0.54
Immigrant, White, <10 yrs	1.11 ± 1.43	0.44	1.07 ± 1.43	0.45
Unk immigration status	0.07 ± 0.05	0.16	0.07 ± 0.05	0.16
Household income	-0.02 ± 0.01	0.13	-0.02 ± 0.01	0.11
High school education	-0.15 ± 0.05	0.00	-0.15 ± 0.05	0.00
Post-secondary education	0.00 ± 0.05	0.98	0.00 ± 0.05	0.99
Unk education status	0.00 ± 0.06	0.95	-0.01 ± 0.06	0.82

Female	0.31 ± 0.02	0.00	0.30 ± 0.02	0.00
Age	-0.24 ± 0.02	0.00	-0.24 ± 0.02	0.00
Weekly activity	0.01 ± 0.01	0.40	0.01 ± 0.01	0.30
Has not quit smoking	0.04 ± 0.12	0.75	0.04 ± 0.12	0.74
Unk smoking quit status	0.20 ± 0.25	0.42	0.18 ± 0.25	0.45
Never smoked	-0.10 ± 0.03	0.00	-0.10 ± 0.03	0.00
Occasional smoker	-0.23 ± 0.06	0.00	-0.24 ± 0.06	0.00
Non-smoker	-0.46 ± 0.04	0.00	-0.47 ± 0.04	0.00
Unk smoking frequency	-0.52 ± 0.41	0.21	-0.54 ± 0.41	0.19
Weekly alcohol	0.01 ± 0.01	0.40	0.01 ± 0.01	0.18
Weekly fruit and veg	-0.03 ± 0.01	0.02	0.01 ± 0.01	0.03

Supplementary References

1. Grade AM, Chan NW, Gajbhiye P, Perkins DJ, Warren PS. Evaluating the use of semi-structured crowdsourced data to quantify inequitable access to urban biodiversity: A case study with eBird. PLoS One. 2022;17(11):e0277223.
2. Matheson FI, Dunn JR, Smith KL, Moineddin R, Glazier RH. Development of the Canadian Marginalization Index: a new tool for the study of inequality. Can J Public Health. 2012;103(8 Suppl 2):S12-6.
3. Markevych I, Schoierer J, Hartig T, Chudnovsky A, Hystad P, Dzhambov AM, et al. Exploring pathways linking greenspace to health: Theoretical and methodological guidance. Environ Res. 2017;158:301-17.
4. Kuras ER, Warren PS, Zinda JA, Aronson MFJ, Cilliers S, Goddard MA, et al. Urban socioeconomic inequality and biodiversity often converge, but not always: A global meta-analysis. Landscape Urban Plann. 2020;198:103799.
5. Wood SN, Goude Y, Shaw S. Generalized additive models for large data sets. J Roy Stat Soc Ser C (Appl Stat). 2015;64(1):139-55.
6. Wood SN. Generalized additive models: an introduction with R. Boca Raton, FL, USA: Chapman and Hall/CRC Press; 2006.
7. Marra G, Wood SN. Practical variable selection for generalized additive models. Comput Stat Data Anal. 2011;55(7):2372-87.
8. Phillips O. Using bootstrap weights with Wes Var and SUDAAN. The Research Data Centres Information and Technical Bulletin. 2004;1(2):6-21.
9. Dormann CF, Elith J, Bacher S, Buchmann C, Carl G, Carré G, et al. Collinearity: a review of methods to deal with it and a simulation study evaluating their performance. Ecography. 2013;36(1):27-46.
10. Amodio S, Aria M, D'Ambrosio A. On concurvity in nonlinear and nonparametric regression models. Statistica. 2015;74(1):85-98.
11. Van Buuren S, Groothuis-Oudshoorn K. mice: Multivariate imputation by chained equations in R. J Stat Softw. 2011;45:1-67.
