# Supplementary material for: Mental health is positively associated with biodiversity in Canadian cities
Source: Commun Earth Environ. 2024 Jun 11;5(1):310. doi: 10.1038/s43247-024-01482-9 (PMC11166573; doi:10.1038/s43247-024-01482-9)
Supplement: Supplementary file 3 — Reporting summary [file 43247_2024_1482_MOESM3_ESM.pdf]

nature portfolio

Corresponding author(s): COMMSENV-23-1923A

Last updated by author(s): Mar 22, 2024

Reporting Summary

Nature Portfolio wishes to improve the reproducibility of the work that we publish. This form provides structure for consistency and transparency in reporting. For further information on Nature Portfolio policies, see our Editorial Policies and the Editorial Policy Checklist.

Statistics

For all statistical analyses, confirm that the following items are present in the figure legend, table legend, main text, or Methods section.

n/a	Confirmed	
☐	☒	The exact sample size (*n*) for each experimental group/condition, given as a discrete number and unit of measurement
☐	☒	A statement on whether measurements were taken from distinct samples or whether the same sample was measured repeatedly
☐	☒	The statistical test(s) used AND whether they are one- or two-sided<br>*Only common tests should be described solely by name; describe more complex techniques in the Methods section.*
☐	☒	A description of all covariates tested
☐	☒	A description of any assumptions or corrections, such as tests of normality and adjustment for multiple comparisons
☐	☒	A full description of the statistical parameters including central tendency (e.g. means) or other basic estimates (e.g. regression coefficient) AND variation (e.g. standard deviation) or associated estimates of uncertainty (e.g. confidence intervals)
☐	☒	For null hypothesis testing, the test statistic (e.g. *F*, *t*, *r*) with confidence intervals, effect sizes, degrees of freedom and *P* value noted<br>*Give P values as exact values whenever suitable.*
☒	☐	For Bayesian analysis, information on the choice of priors and Markov chain Monte Carlo settings
☐	☒	For hierarchical and complex designs, identification of the appropriate level for tests and full reporting of outcomes
☐	☒	Estimates of effect sizes (e.g. Cohen's *d*, Pearson's *r*), indicating how they were calculated

Our web collection on statistics for biologists contains articles on many of the points above.

Software and code

Policy information about availability of computer code

Data collection	CCHS data were accessed at the Carleton University Data Centre, a part of the Canadian Research Data Centre Network (CRDCN). This service is provided through the support of Carleton University, the Canadian Foundation for Innovation, the Canadian Institutes of Health Research, the Social Science and Humanity Research Council, and Statistics Canada.
Data analysis	Data were analyzed using R statistical software version 4.2.1

For manuscripts utilizing custom algorithms or software that are central to the research but not yet described in published literature, software must be made available to editors and reviewers. We strongly encourage code deposition in a community repository (e.g. GitHub). See the Nature Portfolio guidelines for submitting code & software for further information.

Data

Policy information about availability of data

All manuscripts must include a data availability statement. This statement should provide the following information, where applicable:

- Accession codes, unique identifiers, or web links for publicly available datasets
- A description of any restrictions on data availability
- For clinical datasets or third party data, please ensure that the statement adheres to our policy

All secondary data, code, and materials used in the analyses are available here (http://www.github.com/emmajhudgins/equitablecities) – raw data are unavailable due to privacy.

Research involving human participants, their data, or biological material

Policy information about studies with human participants or human data. See also policy information about sex, gender (identity/presentation), and sexual orientation and race, ethnicity and racism.

Reporting on sex and gender	Sex was included as a covariate
Reporting on race, ethnicity, or other socially relevant groupings	White vs non-white and immigration status were included as covariates: (White Canadian-born, non-white Canadian born, white immigrant born outside of Canada (0-9 years in Canada), non-white immigrant born outside of Canada (0-9 years in Canada), white immigrant born outside of Canada (10+ years in Canada), non-white immigrant born outside of Canada (10+ years in Canada)
Population characteristics	We used the following covariates: age, sex, marital status, employment, total household income (ordinal classes treated as a continuous variable), highest level of education attained (treated as a categorical variable), immigration status (White Canadian-born, non-white Canadian born, white immigrant born outside of Canada (0-9 years in Canada), non-white immigrant born outside of Canada (0-9 years in Canada), white immigrant born outside of Canada (10+ years in Canada), non-white immigrant born outside of Canada (10+ years in Canada), and White vs non-White and health behaviors: total daily fruit and vegetable servings consumed per day, smoking status (type of smoker: every day, occasionally, not at all; binary smoking cessation), alcohol consumption, and amount of leisure physical activity.
Recruitment	We used data collected by Statistics Canada as part of the Canadian Community Health Survey
Ethics oversight	Ethics approval not needed for secondary data

Note that full information on the approval of the study protocol must also be provided in the manuscript.

Field-specific reporting

Please select the one below that is the best fit for your research. If you are not sure, read the appropriate sections before making your selection.

☐ Life sciences ☒ Behavioural & social sciences ☐ Ecological, evolutionary & environmental sciences

For a reference copy of the document with all sections, see nature.com/documents/nr-reporting-summary-flat.pdf

Behavioural & social sciences study design

All studies must disclose on these points even when the disclosure is negative.

Study description	We explored how self-reported mental health and self-reported stress relates to bird species diversity and tree species diversity in Canadian postal codes. We used socio-demographic information and health behaviours as covariates. All data were collected as part of the Canadian Community Health Survey from Statistics Canada
Research sample	Subsample of the Canadian Community Health Survey in postal codes that contained bird diversity information (n= 47,623 and self-rated stress n= 48,693)
Sampling strategy	We used Canadian Community Health Survey participants from the 2007 to 2021 in Canadian Metropolitan Areas older than 18 in postal codes with corresponding eBird bird species diversity data
Data collection	All health data were obtained from the Carleton University Data Centre. Canadian Community Health Survey are collected by Statistics Canada https://www23.statcan.gc.ca/imdb/p2SV.pl?Function=getSurvey&SDDS=3226
Timing	Annual survey data collection
Data exclusions	People below the age of 18 not residing in Canadian Metropolitan Areas were excluded to limit the analysis to adults residing in urban areas. Additionally, survey respondents living in postal codes without bird diversity data were excluded.
Non-participation	NA
Randomization	NA

Reporting for specific materials, systems and methods

We require information from authors about some types of materials, experimental systems and methods used in many studies. Here, indicate whether each material, system or method listed is relevant to your study. If you are not sure if a list item applies to your research, read the appropriate section before selecting a response.

Materials & experimental systems

n/a	Involved in the study
☒	☐ Antibodies
☒	☐ Eukaryotic cell lines
☒	☐ Palaeontology and archaeology
☒	☐ Animals and other organisms
☒	☐ Clinical data
☒	☐ Dual use research of concern
☒	☐ Plants

Methods

n/a	Involved in the study
☒	☐ ChIP-seq
☒	☐ Flow cytometry
☒	☐ MRI-based neuroimaging

Plants

Seed stocks	NA
Novel plant genotypes	NA
Authentication	NA
